# Positive family history as a predictor for disease outcomes after radical prostatectomy for nonmetastatic prostate cancer

**DOI:** 10.1080/2090598X.2023.2196911

**Published:** 2023-04-03

**Authors:** Pawel Rajwa, Fahad Quhal, David D’Andrea, Stephan Korn, Patrik Petrov, Takafumi Yanagisawa, Tatsushi Kawada, Reza Sari Motlagh, Hadi Mostafaei, Ekaterina Laukhtina, Abdulmajeed Aydh, Frederik König, Maximilian Pallauf, Benjamin Pradere, Peter Nyirády, Mohammad Abufaraj, Giancarlo Marra, Giorgio Gandaglia, Alberto Briganti, Pierre Karakiewicz, Ding-wei Ye, Martin Haydter, Piotr Chlosta, Eva Comperat, Dmitry Enikeev, Shahrokh F. Shariat

**Affiliations:** a Department of Urology, Medical University of Vienna, Vienna, Austria; b Department of Urology, Medical University of Silesia, Zabrze, Poland; c Department of Urology, King Fahad Specialist Hospital, Dammam, Saudi Arabia; d Department of Urology, The Jikei University School of Medicine, Tokyo, Japan; e Department of Urology, Okayama University Graduate School of Medicine, Okayama, Japan; f Men’s Health and Reproductive Health Research Center, Shahid Beheshti University of Medical Sciences, Tehran, Iran; g Research Center for Evidence Based Medicine, Tabriz University of Medical Sciences, Tabriz, Iran; h Institute for Urology and Reproductive Health, Sechenov University, Moscow, Russia; i Department of Urology, King Faisal Medical City, Abha, Saudi Arabia; j Department of Urology, University Medical Center Hamburg-Eppendorf, Hamburg, Germany; k Department of Urology, Paracelsus Medical University Salzburg, University Hospital Salzburg, Salzburg, Austria; l Department of Urology, Semmelweis University, Budapest, Hungary; m Department of Special Surgery, Division of Urology, Jordan University Hospital, The University of Jordan, Amman, Jordan; n Department of Urology, San Giovanni Battista Hospital, University of Turin, Turin, Italy; o Department of Urology and Division of Experimental Oncology, URI Urological Research Institute, IRCCS San Raffaele Scientific Institute, Milan, Italy; p Cancer Prognostics and Health Outcomes Unit, University of Montreal Health Centre, Montreal, Canada; q Department of Urology, Fudan University Shanghai Cancer Center, Shanghai, China; r Department of Urology, Landesklinikum Wiener Neustadt, Vienna, Austria; s Department of Urology, Jagiellonian University, Krakow, Poland; t Department of Pathology, Medical University of Vienna, Vienna, Austria; u Karl Landsteiner Institute of Urology and Andrology, Vienna, Austria; v Department of Urology, Weill Cornell Medical College, New York, NY, USA; w Department of Urology, University of Texas Southwestern, Dallas, TX, USA; x Department of Urology, Second Faculty of Medicine, Charles University, Prague, Czech Republic; y Division of Urology, Hourani Center for Applied Scientific Research, Al-Ahliyya Amman University, Amman, Jordan

**Keywords:** Family history, prostate cancer, radical prostatectomy, prostate, biochemical recurrence

## Abstract

**Background:**

While family history (FHx) of prostate cancer (PCa) increases the risk of PCa, comparably less is known regarding the impact of FHx on pathologic and oncologic outcomes after radical prostatectomy (RP).

**Methods:**

We retrospectively reviewed our multicenter database comprising 6,041 nonmetastatic PCa patients treated with RP. Patients with a FHx of PCa in one or more first-degree relatives were considered as FHx positive. We examined the association of FHx with pathologic outcomes and biochemical recurrence (BCR) using logistic and Cox regression models, respectively.

**Results:**

In total, 1,677 (28%) patients reported a FHx of PCa. Compared to patients without FHx, those with, were younger at RP (median age of 59 vs. 62 years, *p* < 0.01), and had significantlymore favorable biopsy and RP histopathologic findings. On multivariable logistic regression analysis, positive FHx was associated with extracapsular extension (odds ratio [OR] 0.77, 95% confidence interval [CI] 0.66–0.90, *p* < 0.01; model AUC 0.73) and upgrading (OR 0.70, 95% CI 0.62–0.80, *p* < 0.01; model AUC 0.68). Incorporating FHx significantly improved the AUC of the base model for upgrading (*p* < 0.01). Positive FHx was not associated with BCR in pre- and postoperative multivariable models (*p* = 0.1 and *p* = 0.7); c-indexes of Cox multivariable models were: 0.73 and 0.82, respectively.

**Conclusions:**

We found that patients with clinically nonmetastatic PCa who have positive FHx of PCa undergo RP at a younger age and have more favorable pathologic outcomes. Nevertheless, FHx of PCa did not confer better BCR rates, suggesting that FHx leads to potentially early detection and treatment without impact on BCR.

## Introduction

Men with a family history (FHx) of prostate cancer (PCa) have been shown to harbor an increased risk of developing PCa and at a younger age at diagnosis [1–4]. Indeed, men with a first degree relative have an estimated increase in PCa incidence of approximately 2-fold; those with several relatives affected have an increased risk up to 17-folds [1–3]. Several germline mutations, such as BRCA 1 and BRCA 2, have been found to be associated with an increased risk of PCa as well as more aggressive behavior leading to worse survival outcomes after definitive local treatment [5]. Hereditary PCa has, however, complex multifactorial genetic and epigenetic components, with only a small proportion of patients having identifiable germline mutations [6]. Nevertheless, current EAU guidelines recommend early germline testing in men with positive FHx of PCa [7]. Overtime, with the widespread uptake of the PSA testing, positive FHx has become more unclear with a need for thorough evaluation to improve our understanding of the disease [6].

Radical prostatectomy (RP) is a broadly utilized and effective treatment option for clinically nonmetastatic (cM0) PCa [8,9]. Approximately 6–30% of PCa patients, in industrialized countries, report first-degree relatives affected by PCa more than half of these men with cM0 PCa eventually undergo RP [10,11]. In general, previous reports have shown either no association of positive FHx with pathologic features [12] or even a more favorable pathologic characteristics in patients with PCa FHx [13]. A recent meta-analysis, analyzing data from four studies, found no significant association between positive FHx and biochemical recurrence (BCR), albeit authors noted significant heterogeneity across studies [11]. Indeed, the included studies reported a protective [13], neutral [14,15], and negative [12] association of PCa FHx.

Considering the high prevalence of PCa patients with affected relatives and the recent advances in identifying germline alterations, there is a need to further explore the prognostic value of positive FHx in patients with cM0 PCa. To address this, we performed a study analyzing the predictive and prognostic value of positive PCa FHx in patients who underwent RP for cM0 PCa.

### Materials and methods

We analyzed our database of 6,041 patients who underwent RP for cM0 PCa at five centers [16]. All included patients had available data on FHx. FHx was considered positive if they had one or more first-degree relatives with a history of PCa. All patients had clinically nonmetastatic PCa on conventional imaging (bone scan and computed tomography). None of the patients received neoadjuvant hormonal therapy or adjuvant radiotherapy. All patients underwent RP with or without pelvic lymph node dissection (PLND) depending on guidelines recommendations at the time and physician preference. Pathologic examination was performed by expert genitourinary pathologists at each center. Pathologic stage was evaluated according to the 2009 American Joint Committee on Cancer TNM staging system. For the analyzes we used the 2015 Grade group (GG) classification, instead of initially implemented International Society of Urological Pathology 2005 Gleason Score (GS) [17]. In general, after RP, patients underwent physical examination and prostate-specific antigen (PSA) testing every three months for the first year after surgery and then semiannually from the second year. Follow-up was calculated from the date of RP. We considered BCR as two PSA readings of>0.2 ng/ml [18,19].

### Statistical analyses

Correlations between clinicopathologic characteristics and FHx were assessed using the Wilcoxon rank sum or Pearson’s Chi-squared test, as appropriate. We tested the association of FHx with pathologic outcomes and biochemical recurrence (BCR) using logistic and Cox regression models, respectively. Pathologic outcomes of interests were: extracapsular extension (pT3a), lymph node involvement (pN ≥ 1), upgrading at RP and adverse pathology (defined as pT ≥ 3 and/or pN ≥ 1 and/or GG ≥ 4 and/or positive surgical margins). The receiver operating (ROC) curve with area under the curve (AUC) were employed to evaluated models’ discrimination. Decision curve analysis (DCA) was used to assess the clinical benefit of the implementation of FHx. Kaplan-Meier analysis with log-rank test was applied for pairwise comparisons of BCR free survival. Multivariable Cox regression models were constructed to test the association between FHx and the risk of BCR with Harrell's concordance index (c-index) testing the discrimination ability [20]. Considering baseline differences between patients with positive and negative FHx we further used a 1:1 propensity-score-matched analysis. The use of the propensity score method reduces the customary bias associated with the conventional multivariable modeling approach. The variables adjusted for were cT stage, biopsy GG, diabetes mellitus, and smoking status. All tests of significance were 2-sided, and *p* < 0.05 was considered statistically significant. All statistics were done in R Version 4.0 (R Foundation for Statistical Computing, Vienna, Austria, 2020).

## Results

### Clinicopathologic findings

In total, 1,677 (28%) patients reported positive FHx. The cohort characteristics are summarized in Table 1. The median age at RP was 61 years (interquartile range [IQR] 57–66), and patients were followed for a median of 44 months (IQR 31–57) after RP. Patients with positive FHx were significantly younger at RP (median: 59 years [IQR 55–65] vs. 62 years [IQR 58–67], *p* < 0.001). Patients with positive and negative FHx did not differ in terms of median PSA (*p* = 0.9). Patients with positive FHx were more often diagnosed with GG 1 disease at biopsy (65% vs. 59%, *p* < 0.001) and at RP (39% vs. 29%, *p* < 0.001). Patients with positive FHx were less likely to harbour extraprostatic extension (18% vs. 24%, *p* < 0.001) and seminal vesicle invasion at RP (3.3% vs. 7%, *p* < 0.001).

**Table 1.** Basic clinicopathologic features of 6,041 patients treated with radical prostatectomy for nonmetastatic prostate cancer.

	Overall	Family history		
Characteristic	*N* = 6,041	negative, *N* = 4,364	positive, *N* = 1,677	p-value
Family history (positive)	1,677 (28%)			
Age, median (IQR)	61 (57–66)	62 (58–67)	59 (55–65)	<0.001
Smoker				0.001
Ever (%)	1,613 (27%)	1,172 (27%)	441 (26%)	
Former (%)	2,131 (35%)	1,482 (34%)	649 (39%)	
Current(%)	2,297 (38%)	1,710 (39%)	587 (35%)	
DM, n (%)	598 (9.9%)	427 (9.8%)	171 (10%)	0.6
BMI (IQR)	28.0 (25.0, 32.0)	28.0 (25.0, 32.0)	28.0 (25.0, 32.0)	0.3
PSA [ng/ml] (IQR)	6.0 (4.0, 9.0)	6.0 (4.0, 9.0)	6.0 (4.0, 9.0)	0.091
cT stage				0.8
1	4,299 (71%)	3,104 (71%)	1,195 (71%)	
2	1,714 (28%)	1,238 (28%)	476 (28%)	
≥3	28 (0.5%)	22 (0.5%)	6 (0.4%)	
Biopsy GG				<0.001
1	3,660 (61%)	2,565 (59%)	1,095 (65%)	
2	1,364 (23%)	1,019 (23%)	345 (21%)	
3	637 (11%)	488 (11%)	149 (8.9%)	
4	278 (4.6%)	211 (4.8%)	67 (4.0%)	
5	102 (1.7%)	81 (1.9%)	21 (1.3%)	
RP GG				<0.001
1	1,932 (32%)	1,271 (29%)	661 (39%)	
2	2,187 (36%)	1,598 (37%)	589 (35%)	
3	1,512 (25%)	1,169 (27%)	343 (20%)	
4	202 (3.3%)	150 (3.4%)	52 (3.1%)	
5	208 (3.4%)	176 (4.0%)	32 (1.9%)	
GG upgrading	2,539 (42%)	1,912 (44%)	627 (37%)	<0.001
LVI	693 (11%)	509 (12%)	184 (11%)	0.5
pN+	116 (1.9%)	93 (2.1%)	23 (1.4%)	0.054
pT3a	1,367 (23%)	1,061 (24%)	306 (18%)	<0.001
pT3b	361 (6.0%)	305 (7.0%)	56 (3.3%)	<0.001
PSM	794 (13%)	595 (14%)	199 (12%)	0.069
Follow-up	44 (31, 57)	44 (31, 56)	45 (32, 60)	0.017
BCR	761 (13%)	580 (13%)	181 (11%)	0.009

Statistics presented: Median (IQR), n (%); statistical tests performed: Wilcoxon rank sum test; Pearson’s Chi-squared test.

Abbreviations: BCR, biochemical recurrence; BMI, body mass index; DM, diabetes mellitus; FHx, family history; GG, Gleason Grade; LVI, lymphovascular invasion; PSA, prostate-specific antigen; PSM, positive surgical margin; RP, radical prostatectomy.

### Adverse pathologic findings

**Figure 1.**
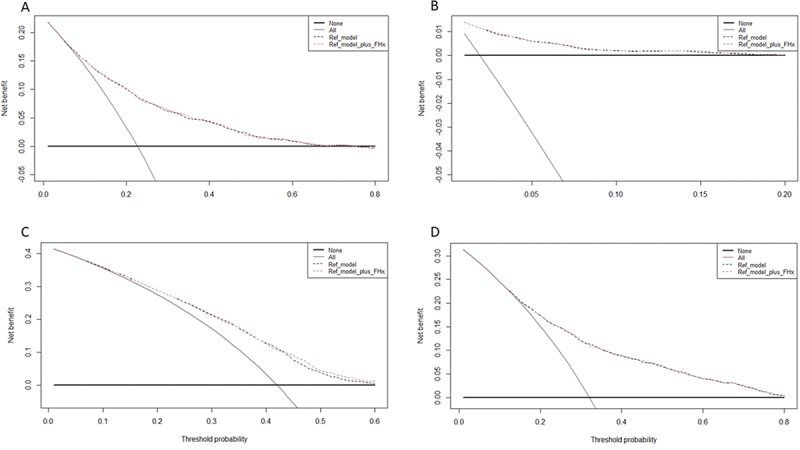
Decision curve analysis (DCA) for the net-benefit of the preoperative family history (FHx) based on the preoperative reference model for the prediction of: (A) extracapsular extension (pT3a), (B) lymph node involvement (pN ≥ 1) (C) upgrading, (D) adverse pathology at radical prostatectomy (RP).

In univariable analyses, positive FHx was associated with a lower risk of pT3a disease (odds ratio [OR], 0.69, 95% CI 0.60–0.80, *p* < 0.001), GG upgrading at RP (OR 0.77, 95% CI 0.68–0.86, *p* < 0.001) and adverse pathology (OR 0.75, 95% CI 0.66–0.85, *p* < 0.001), but not pN ≥ 1 (OR 0.64, 95% CI 0.40–1.01, *p* = 0.06). Upon adjustment for the effects of age, preoperative PSA, cT stage and biopsy GG (Table 2), positive FHx remained independently associated with a lower risk of pT3a disease (OR 0.76, 95% CI 0.65–0.89, *p* = 0.001), GG upgrading at RP (OR 0.70, 95% CI 0.62–0.79, *p* < 0.001), and adverse pathology (OR 0.82, 95% CI 0.71–0.94, *p* = 0.004). Incorporating FHx significantly improved the AUC of the baseline model, comprising age, preoperative PSA, cT stage and biopsy GG, for the prediction of GG upgrading (*p* = 0.002). On decision curve analysis (DCA), there was no clinical net benefit of the (Figure 1). On exploratory multivariable analysis among GG1 patients (*n* = 1,952) only, positive FHx was associated with a lower risk of GG upgrading (OR 0.66, 95% CI 0.57–0.76, *p* < 0.001), but not with the risk of pT3a (*p* = 0.16), pN1 ≥ 1 (*p* = 0.92), or adverse pathology (*p* = 0.17).

**Table 2.** Multivariable logistic regression analyses assessing the association of family history (FHx) with adverse surgical features at radical prostatectomy (RP) in 6,041 patients with clinically nonmetastatic prostate cancer.

	pT3a			N			Upgrading at RP			Adverse pathology		
Characteristic	OR	95% CI	p-value	OR	95% CI	p-value	OR	95% CI	p-value	OR	95% CI	p-value
FHx (positive vs. negative)	0.76	0.65–0.89	0.001	0.77	0.48–1.26	0.298	0.70	0.62–0.79	<0.001	0.82	0.71–0.94	0.004
Age	1.02	1.01–1.03	0.001	1.03	1.00–1.06	0.079	1.01	1.00–1.02	0.060	1.01	1.00–1.02	0.019
PSA	1.07	1.06–1.08	<0.001	1.04	1.02–1.06	<0.001	1.04	1.03–1.06	<0.001	1.08	1.07–1.09	<0.001
cT ≥ 2 stage	2.34	2.04–2.68	<0.001	3.00	2.00–4.49	<0.001	1.62	1.43–1.83	<0.001	2.10	1.85–2.38	<0.001
Biopsy GG group												
GG group 1	Ref.	Ref.		Ref.	Ref.		Ref.	Ref.		Ref.	Ref.	
GG group 2	2.77	2.37–3.24	<0.001	7.16	3.49–14.71	<0.001	0.55	0.49–0.63	<0.001	2.04	1.77–2.35	<0.001
GG group 3	4.43	3.65–5.37	<0.001	13.53	6.55–27.94	<0.001	0.11	0.09–0.15	<0.001	3.23	2.69–3.88	<0.001
GG group 4	5.43	4.16–7.09	<0.001	22.24	10.35–47.80	<0.001	0.19	0.14–0.26	<0.001	3.79	2.92–4.93	<0.001
GG group 5	12.15	7.73–19.10	<0.001	55.52	24.73–124.64	<0.001	N/A	N/A	N/A	6.94	4.41–10.90	<0.001
	AUC (full model): 0.763 AUC (model without FHx): 0.762 *p* = 0.37			AUC (full model): 0.880 AUC (model without FHx): 0.880 *p* = 0.69			AUC (full model): 0.683 AUC (model without FHx): 0.675 *p* = 0.002			AUC (full model): 0.722 AUC (model without FHx): 0.721 *p* = 0.419		

Adverse pathology (pT ≥ 3 and/or pN ≥ 1 and/or GG ≥ 4 and/or PSM).

Abbreviations: CI, Confidence Interval; GG, Gleason Grade; OR, Odds Ratio; PSA, prostate-specific antigen; RP, radical prostatectomy; SII, Systemic Immune-inflammation Index.

### Biochemical recurrence

**Figure 2.**
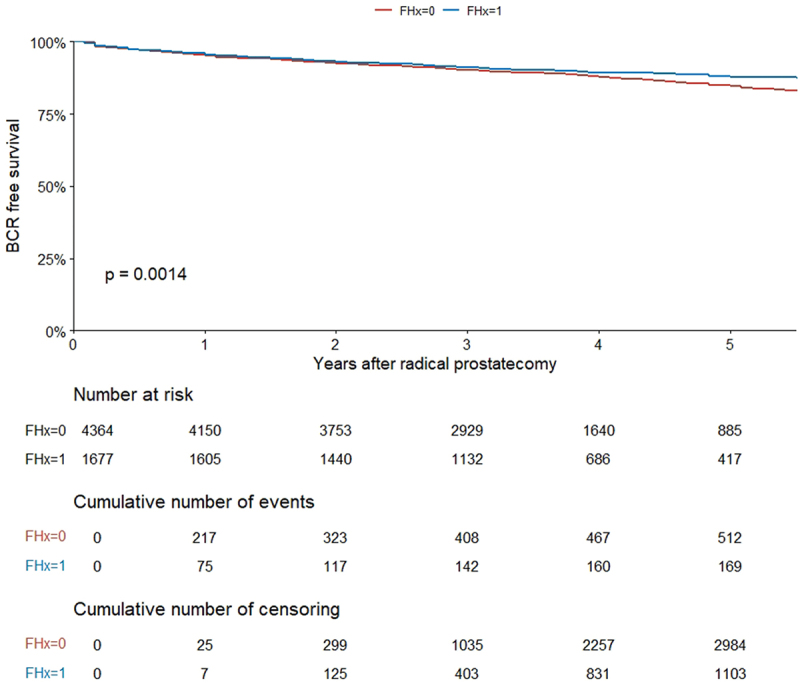
Kaplan-Meier analysis for biochemical recurrence (BCR) free survival stratified by family history.

In total, 181 patients (11%) with positive FHx experienced BCR compared to 580 (13%) with negative FHx (*p* = 0.009). Kaplan Meier estimates revealed that patients with a positive FHx were at lower risk of BCR compared to those with a negative FHx (*p* = 0.0014; Figure 2). At 6 months and 3 years BCR-free survival rates for patients with a positive vs. negative FHx were 97.1% (95% CI 96.3–97.9) vs. 97.0% (95% CI 96.4–97.5) and 91.1% (95% CI 89.7–92.5) vs. 90.2% (95% CI 89.3–91.1), respectively. On univariable Cox regression model, positive FHx was significantly associated with lower risk of BCR (HR 0.76, (95% CI 0.65–0.90), *p* = 0.001) (Table 3). On multivariable Cox regression analyses controlled for established pre- or postoperative confounders, positive FHx was not significantly associated with BCR (HR 0.87, 95% CI 0.73–1.03, *p* = 0.103 and HR 1.03, 95% CI, 0.87–1.22, *p* = 0.734, respectively) (Table 3). The AUCs for the preoperative and postoperative clinical models were 74.5% and 81.7%, respectively. We performed propensity-score matching to compare patients with different FHx status (1677 positive and 1677 negative FHx), and found no impact of FHx on the risk of BCR in the univariable model (HR 1.05, 0.85–1.30, *p* = 0.66) (Table 3).

**Table 3.** Multivariable cox regression models analyzing the impact of family history on BCR-free survival in 6,041 patients treated with radical prostatectomy for clinically nonmetastatic prostate cancer: (a) preoperative model (b) postoperative model.

a)						
Preoperative model						
	Univariable			Multivariable		
Characteristic	HR	95% CI	p-value	HR	95% CI	p-value
Family history (positive vs negative)	0.76	0.65–0.90	0.001	0.87	0.73–1.03	0.103
Age	1.02	1.01–1.03	<0.001	1.01	1.00–1.02	0.053
PSA	1.06	1.05–1.06	<0.001	1.05	1.04–1.05	<0.001
cT ≥ 2 stage	2.39	2.07–2.75	<0.001	1.81	1.56–2.09	<0.001
Biopsy GG group						
GG group 1	Ref.	Ref.		Ref.	Ref.	
GG group 2	1.94	1.62–2.33	<0.001	1.71	1.42–2.05	<0.001
GG group 3	3.19	2.61–3.89	<0.001	2.56	2.09–3.14	<0.001
GG group 4	4.78	3.74–6.11	<0.001	3.76	2.94–4.83	<0.001
GG group 5	8.11	5.87–11.22	<0.001	4.84	3.43–6.83	<0.001
				c-index (full model): 74.4 c index (model without FHx): 74.5		
b)						
Postoperative model						
	Univariable			Multivariable		
Characteristic	HR	95% CI	p-value	HR	95% CI	p-value
FHx (positive vs. negative)	0.76	0.65–0.90	0.001	1.03	0.87–1.22	0.7337
Age	1.02	1.01–1.03	<0.001	1.00	0.99–1.02	0.4040
PSA	1.06	1.05–1.06	<0.001	1.04	1.03–1.04	<0.001
pT ≥ 3	3.36	3.08–3.67	<0.001	2.03	1.82–2.25	<0.001
pN ≥ 1	15.17	12.10–19.03	<0.001	3.15	2.40–4.13	<0.001
PSM	3.76	3.23–4.37	<0.001	1.97	1.68–2.31	<0.001
RP GG group						
GG group 1	Ref.	Ref.		Ref.	Ref.	
GG group 2	1.57	1.24–1.99	<0.001	1.24	0.98–1.57	0.0763
GG group 3	4.06	3.27–5.05	<0.001	2.34	1.85–2.94	<0.001
GG group 4	9.39	7.10–12.42	<0.001	3.35	2.47–4.54	<0.001
GG group 5	13.90	10.57–18.27	<0.001	2.85	2.03–3.99	<0.001
				C-index (full model): 81.7 C-index (full model without FHx): 81.7		

Abbreviations: CI, confidence interval; FHx, family history; GG, Gleason Grade; HR, hazard ratio; PSA, prostate-specific antigen; PSM, positive surgical margin; RP, radical prostatectomy; SII, Systemic Immune-inflammation Index.

## Discussion

The management of PCa has rapidly evolved over the years, with an increased emphasis on patients-centered tailored strategies. Recently, various genomic assays and novel imaging modalities have been incorporated in our armamentarium to refine clinical models with the goals of improving patient selection and treatment choices. Despite these progresses, we still do not have a full understanding of the relevance of certain readily available clinical information such as the FHx of PCa. The predictive and prognostic value of this feature may have also changed over the years with the widespread application of the PSA-testing leading to a sharp increase in the proportion of men affected with PCa.

In our study, we analyzed a large series of clinically nonmetastatic PCa patients treated with RP with curative intent. We found that patients with positive FHx underwent RP at a younger age and had more favorable pathologic outcomes. These findings can be explained by patients’ greater awareness of the disease leading to a more proactive PSA-testing, increased scrutiny from younger ages and fear associated with delayed treatment. Moreover, major guidelines recommend earlier PSA testing in patients with a positive FHx, which can further translate into earlier disease detection and subsequent treatment [7]. Interestingly, a recent report from EMBRACE study suggests a higher PCa incidence among BRCA 1/2 mutation carriers with positive FHx than those with negative FHx [21]. Furthermore, patients with germline mutations had more aggressive disease at diagnosis, which was not observed in our cohort [21,22]. While we lack strong evidence on the utility of PCa screening among germline mutations carriers, we also don’t know if PSA testing and treatment in a case of PCa detection in patients with a positive FHx improves survival or not [23]. Indeed, it may only lead to a lead time bias. Our findings suggest a possible risk of overdiagnosis or too early diagnosis with quality of life loss due to early treatment in patients with positive FHx; presently more appropriate management in those patients would initial active surveillance and personalized approach based on more detailed imaging and biomarkers [24–26]. Recently, Schneider et al. found that positive FHx does not seem to impact disease trajectory during active surveillance [27], and there are conflicting results regarding the role of certain germline mutations [28,29]. In our study, we report that positive FHx had a protective effect for GG upgrading at RP in all patients and among those with GG = 1 at biopsy.

Although we demonstrated a lower BCR risk in patients with positive FHx in univariable analysis, this association was not maintained after adjustment for the effect of established pre- and postoperative clinicopathologic factors. These findings are in line with the studies of Thalgott et al. [15] and Roehl et al. [14], who failed to find an association between positive FHx and BCR after RP. On the other hand, one study [12] reported an over 2-fold reduction BCR-free survival in patients with a positive FHx; however, the sample size (*n* = 506) and short follow-up (median 21 months) may limit these conclusions. In recent retrospective studies, BRCA1/2 germline mutations were associated with significantly worse outcomes after definitive local therapy for PCa with a median cancer-specific survival of 8.6 years compared to 15.7 years in non-carriers (*p* < 0.01) [30]. Comparably, little is known regarding the prognostic value of other germline mutations and single nucleotide polymorphisms (SNPs) [31]. Taking into account our findings that showed no association between FHx and BCR; as well as other reports on the relevance of several genomic features in PCa, and the multifactorial aetiology of hereditary PCa, there is a clinical need for genetic counseling and further studies [32]. Nevertheless, we do not have personalized treatment options for patients with specific genetic aberrations and early stage PCa. Thus, until we have broadly available genetic screening programs and evidence-based optimized treatment strategies among patients with hereditary PCa, FHx should not guide treatment decision-making in PCa.

There are several limitations to our study. First of all, the retrospective design of the study. Second, lack information of other important oncologic endpoints such as metastasis-free or cancer-specific survival; the follow-up of our study was too short to assess these endpoints. Third, no central pathology review was performed. Fourth, FHx was self-reported by patients, and we could not exclude undetected or unknown PCa in first-degree relatives. Moreover, we did not obtain data on the number or age at diagnosis of affected relatives. Finally, patients did not undergo genetic testing, so we could not determine the underlying genetic component of their hereditary PCa. We believe future prospective studies among patients with positive FHx, based on individual genetic aberration, are needed to determine the prognostic relevance of the different types of hereditary PCa.

## Conclusions

In this large cohort of men treated with RP for clinically nonmetastatic PCa, we found that patients with positive FHx of PCA underwent RP at a younger age and had more favorable pathologic outcomes compared to their non-FHx counterparts. Nevertheless, positive FHx did not have a statistically significant impact on BCR-free survival. Taken together, our findings suggest that positive FHx, without precise genetic testing should not influence treatment decision-making in clinically cM0 PCa treated with RP. Moreover, given the high proportion of men affected with PCa, FHx may not have the predictive/prognostic value as expected in the past, before the widespread use of PSA-testing leading to surge in PCa detection.
